# Supplementary material for: Microbiota Dynamics of Mechanically Separated Organic Fraction of Municipal Solid Waste during Composting
Source: Microorganisms. 2021 Sep 3;9(9):1877. doi: 10.3390/microorganisms9091877 (PMC8468692; doi:10.3390/microorganisms9091877)
Supplement: Supplementary file 1 [file microorganisms-09-01877-s001.zip › microorganisms-1323412-supplementary.pdf]

**Table S1.** Morphological composition of ms-OFMSW (fraction <70 mm) and the heat capacity of its components

| № | Components | Content*, % (m/m) | Heat capacity kJ kg$^{-1}$ °C$^{-1}$ [19] | Weighted average heat capacity, kJ kg$^{-1}$ °C$^{-1}$ |
|---|---|---|---|---|
| 1. | Food waste | 55.87 ± 2.43 | 1.99 | 1.11 |
| 2. | Waste paper | 8.56 ± 0.37 | 1.40 | 0.12 |
| 3. | Plastic | 8.27 ± 0.79 | 1.73 | 0.14 |
| 4. | Stone, sand, ceramics | 7.77 ± 0.21 | 1.03 | 0.08 |
| 5. | Combined materials (Tetra Pak, etc.) | 6.85 ± 0.31 | 1.36 | 0.09 |
| 6. | Glass | 6.16 ± 0.24 | 0.66 | 0.04 |
| 7. | Wood | 4.48 ± 0.22 | 1.61 | 0.07 |
| 8. | Metal | 0.69 ± 0.03 | 0.93 | 0.01 |
| 9. | Textile | 0.56 ± 0.02 | 1.29 | 0.01 |
| 10. | Other (fraction less than 15 mm) | 0.79 ± 0.03 | 1.33 | 0.01 |
| | Total: | 100.00 | | 1.68 |

* - average value ± standard deviation of three replicates

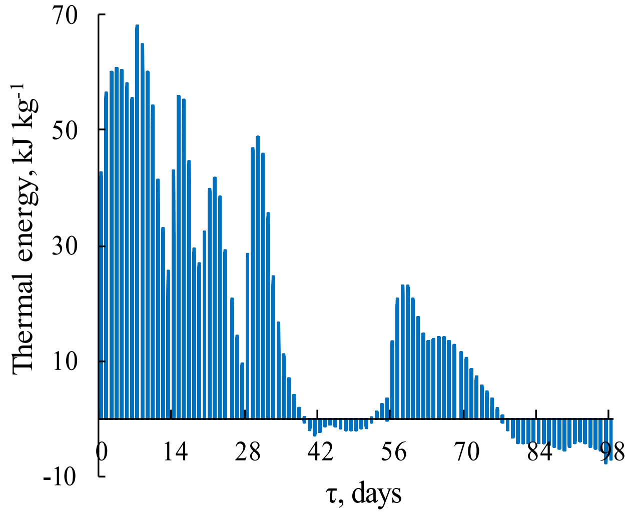


**Figure S1.** Heat release during composting

**Table S2.** Pearson correlations between microbiological and environmental variables: $r_{crit}$ (f=4, $\alpha$=0.05) = 0.81 (Strode, Brokaw, 2015)

| | $CO_2$, g kg$^{-1}$ day$^{-1}$ | T, °C | $NH_3$, mg kg$^{-1}$ day$^{-1}$ | EC, µS cm$^{-1}$ | pH | OM, % | C, % | N, % | C/N | GI, % | NI | Water content, % | Heat release, kJ kg$^{-1}$ |
|---|---|---|---|---|---|---|---|---|---|---|---|---|---|
| *Bacillus* | 0.73 | 0.70 | 0.92 | -0.37 | 0.67 | -0.24 | -0.24 | -0.54 | -0.32 | 0.06 | -0.13 | -0.89 | 0.87 |
| *uncultured Bacillaceae* | -0.21 | 0.64 | 0.11 | -0.80 | 0.04 | 0.97 | 0.97 | -0.20 | 0.82 | -0.79 | -0.47 | -0.46 | 0.38 |
| *Ureibacillus* | -0.30 | -0.91 | 0.98 | -0.22 | 1.00 | n.d. | n.d. | n.d. | n.d. | -0.64 | -0.26 | 0.67 | 0.71 |
| *Aeribacillus* | 0.95 | -0.96 | 0.81 | 0.85 | 0.92 | n.d. | n.d. | n.d. | n.d. | 0.75 | -0.56 | 0.01 | 0.98 |
| *Novibacillus* | -0.19 | 0.56 | 0.04 | -0.50 | 0.01 | 0.76 | 0.76 | -0.67 | 1.00 | -0.59 | 0.11 | -0.55 | -0.17 |
| *Streptomyces* | 0.42 | 0.23 | -0.54 | 0.11 | -0.62 | 0.47 | 0.47 | 0.95 | -0.91 | 0.33 | -0.12 | 0.07 | -0.18 |
| *Tepidimicrobium* | 0.96 | 0.47 | 0.79 | -0.26 | 0.45 | 0.09 | 0.09 | n.d. | n.d. | 0.09 | 0.09 | -0.74 | 0.93 |
| *Thermobifida* | 0.28 | 0.62 | 0.11 | -0.69 | 0.15 | 0.92 | 0.92 | -0.72 | 1.00 | -0.79 | 0.02 | -0.80 | 0.23 |
| *Geobacillus* | -0.47 | -0.82 | 1.00 | -0.40 | 0.99 | n.d. | n.d. | n.d. | n.d. | -0.77 | -0.07 | 0.52 | 0.56 |
| *Paenibacillus* | -0.94 | 0.84 | 0.41 | -0.59 | -0.85 | 0.28 | 0.28 | 0.75 | -0.09 | -0.22 | -1.00 | 0.23 | -0.29 |
| *Planifilum* | 0.37 | 0.24 | 0.96 | -0.52 | 0.41 | 0.62 | 0.62 | -1.00 | 0.78 | -0.62 | -0.09 | -0.69 | 0.78 |
| *Saccharomonospora* | -0.77 | -0.27 | -0.64 | 0.19 | 0.35 | -0.10 | -0.11 | -0.79 | 1.00 | -0.05 | 0.45 | 0.29 | -0.71 |
| *Oceanobacillus* | -0.20 | -0.28 | -0.46 | -0.04 | 0.74 | 0.33 | 0.33 | -0.87 | 0.97 | -0.31 | 0.39 | -0.08 | -0.14 |
| *Vulgatibacter* | -0.90 | -0.83 | -0.58 | 0.85 | 0.43 | -0.98 | -0.98 | n.d. | n.d. | 0.67 | 0.50 | 0.78 | -0.96 |
| SO134 | -0.59 | -0.84 | -0.72 | 0.62 | 0.76 | -0.54 | -0.54 | -0.88 | 0.33 | 0.33 | 0.69 | 0.52 | -0.70 |
| *Thermomyces* | 0.31 | 0.21 | -0.37 | 0.31 | 0.53 | -0.48 | -0.48 | -0.70 | -0.30 | 0.50 | 0.31 | -0.28 | -0.09 |
| *Penicillium* | 0.15 | -0.10 | 0.75 | -0.27 | -0.04 | 0.82 | 0.82 | 0.92 | 0.78 | -0.28 | -0.37 | -0.02 | 0.69 |
| *Microascus* | -0.17 | 0.41 | 0.29 | -0.08 | 0.15 | -0.70 | -0.70 | 0.64 | -0.91 | 0.08 | 0.02 | -0.26 | -0.13 |
| *Aspergillus* | 0.37 | -0.05 | 0.76 | -0.17 | 0.23 | 0.06 | 0.06 | -0.44 | 0.23 | -0.11 | -0.15 | -0.25 | 0.77 |
| Chao1 prokaryotes | -0.06 | -0.11 | -0.25 | 0.79 | 0.24 | -0.81 | -0.81 | 0.36 | -0.96 | 0.88 | 0.20 | 0.23 | -0.37 |
| Shannon prokaryotes | -0.53 | -0.41 | -0.72 | 0.78 | -0.06 | -0.77 | -0.77 | 0.50 | -0.96 | 0.67 | 0.16 | 0.64 | -0.79 |
| Chao1 fungi | 0.27 | -0.10 | 0.72 | -0.36 | -0.21 | 0.66 | 0.66 | 0.55 | 0.39 | -0.39 | -0.34 | -0.05 | 0.68 |
| Shannon fungi | -0.01 | -0.28 | 0.58 | -0.21 | -0.27 | 0.39 | 0.39 | 0.70 | 0.21 | -0.34 | -0.32 | 0.17 | 0.46 |

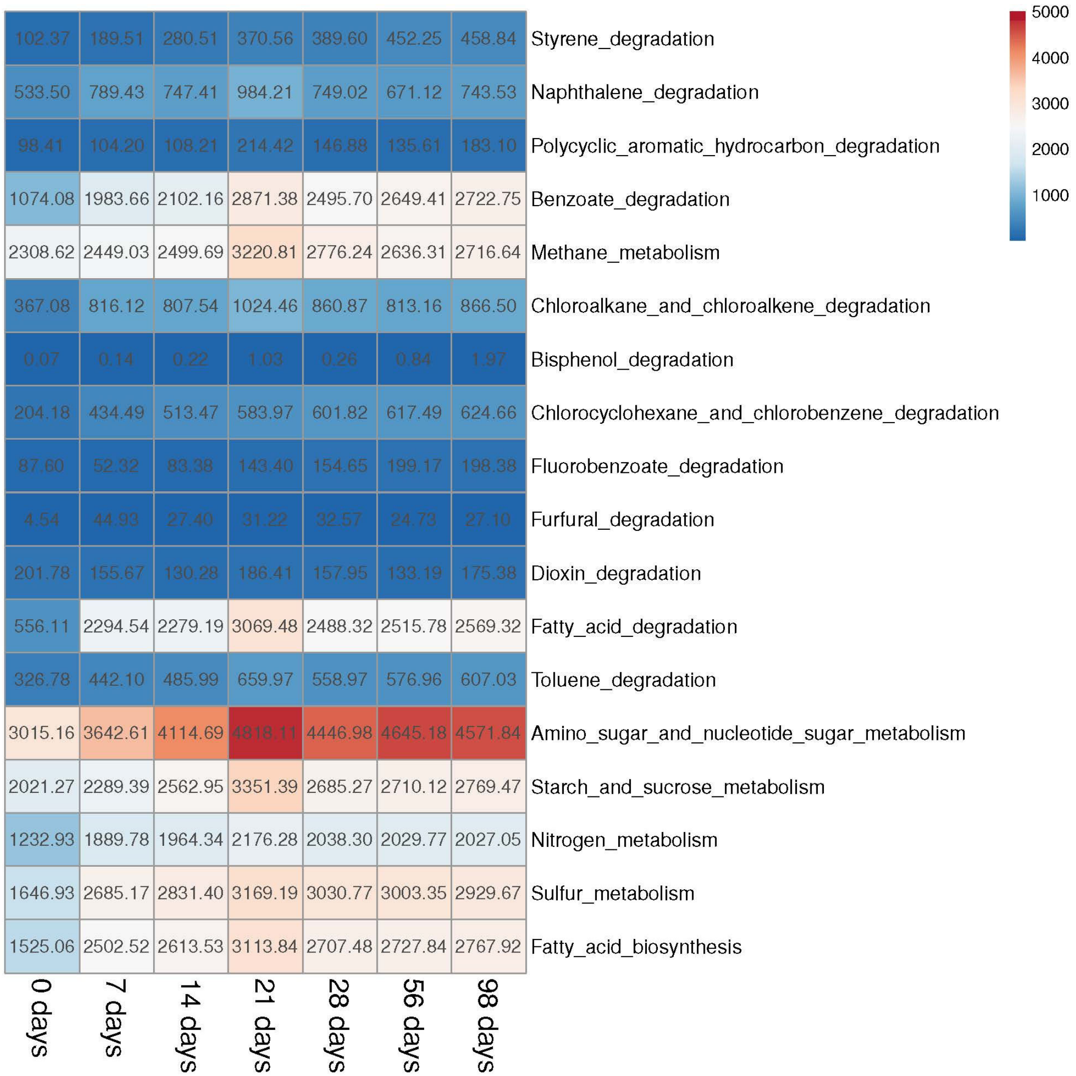


**Figure S2.** The heat map of prokaryotic microbiota functional profiles on day 0, 7, 14, 21, 28, 56, and 98 of composting. The number of genes related to particular metabolic pathway is shown by color.

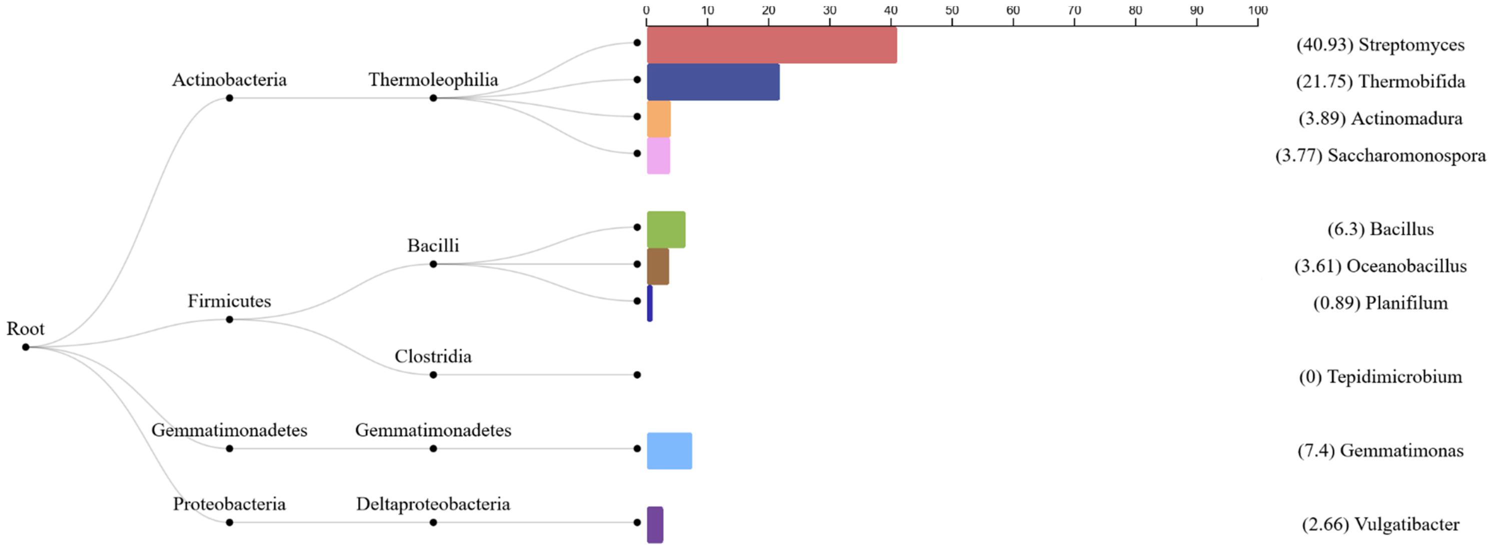
0
10
20
30
40
50
60
70
80
90
100
Root
Actinobacteria
Thermoleophilia
Firmicutes
Bacilli
Clostridia
Gemmatimonadetes
Gemmatimonadetes
Proteobacteria
Deltaproteobacteria
(40.93) Streptomyces
(21.75) Thermobifida
(3.89) Actinomadura
(3.77) Saccharomonospora
(6.3) Bacillus
(3.61) Oceanobacillus
(0.89) Planifilum
(0) Tepidimicrobium
(7.4) Gemmatimonas
(2.66) Vulgatibacter

(a)

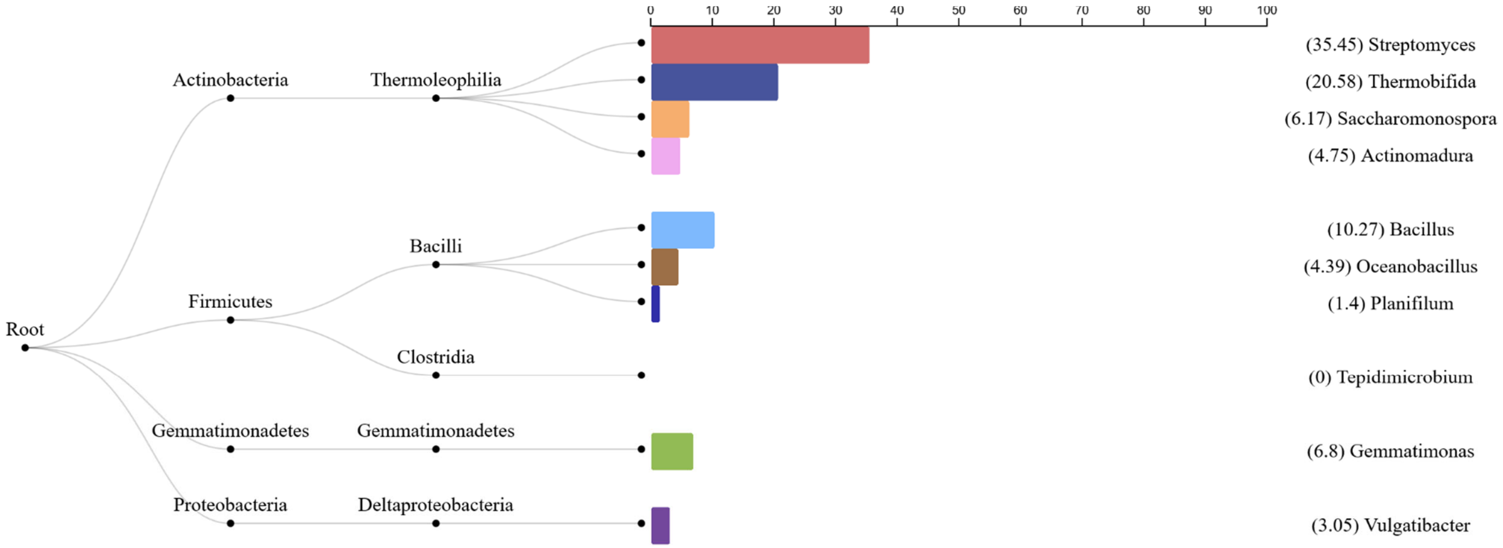
0
10
20
30
40
50
60
70
80
90
100
Root
Actinobacteria
Thermoleophilia
Firmicutes
Bacilli
Clostridia
Gemmatimonadetes
Gemmatimonadetes
Proteobacteria
Deltaproteobacteria
(35.45) Streptomyces
(20.58) Thermobifida
(6.17) Saccharomonospora
(4.75) Actinomadura
(10.27) Bacillus
(4.39) Oceanobacillus
(1.4) Planifilum
(0) Tepidimicrobium
(6.8) Gemmatimonas
(3.05) Vulgatibacter

(b)

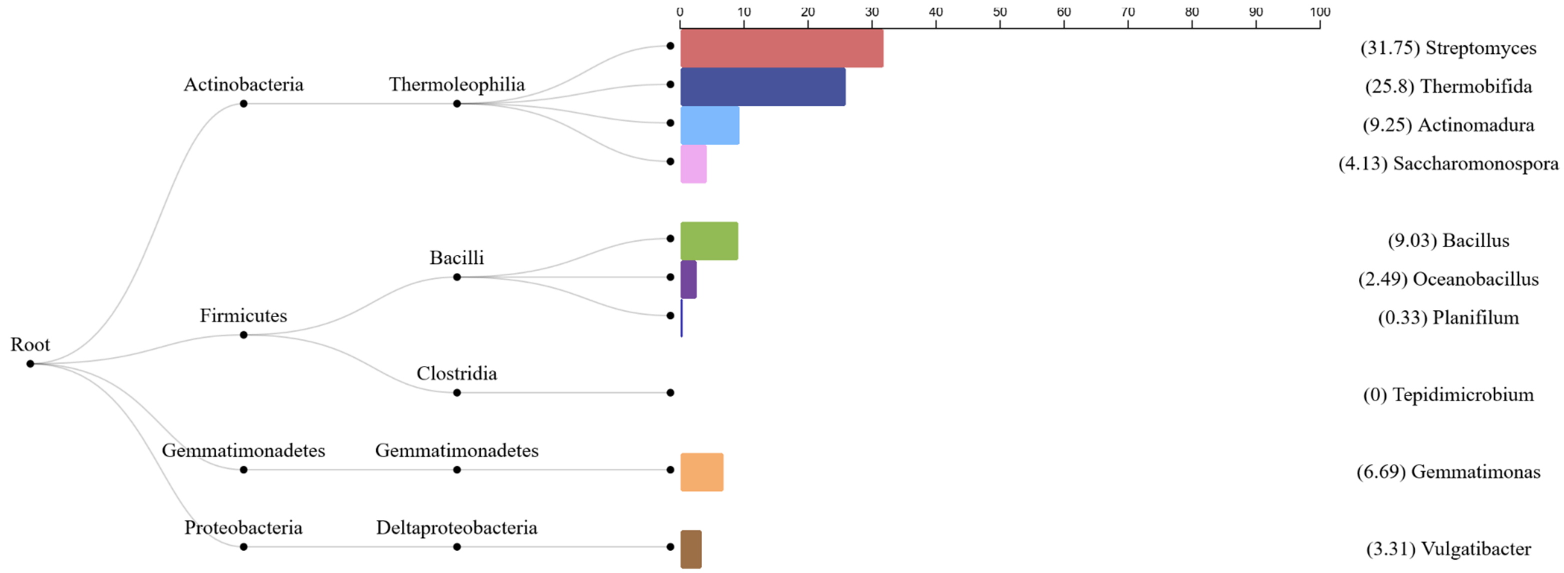


(c)

**Figure S3.** Dendrograms showing key microorganisms and their phylogenetic lines involved in metabolism of: (a) starch and sucrose, (b) methane, (c) fatty acid degradation.
